# Antimicrobial Stewardship: From Bedside to Theory. Thirteen Examples of Old and More Recent Strategies from Everyday Clinical Practice

**DOI:** 10.3390/antibiotics9070398

**Published:** 2020-07-10

**Authors:** Stefano Di Bella, Bojana Beović, Massimiliano Fabbiani, Michael Valentini, Roberto Luzzati

**Affiliations:** 1 Department of Medical, Surgical and Health Sciences, Trieste University, 34127 Trieste, Italy; roberto.luzzati@asugi.sanita.fvg.it; 2 Department of Infectious Diseases, University Medical Centre Ljubljana, 1000 Ljubljana, Slovenia; bojana.beovic@kclj.si; 3 Infectious Disease Unit, Azienda Ospedaliero-Universitaria Senese, 53100 Siena, Italy; massimiliano.fabbiani@gmail.com; 4 Azienda Sanitaria Universitaria Giuliano Isontina, 34128 Trieste, Italy; michael.valentini@asugi.sanita.fvg.it

**Keywords:** antimicrobial stewardship, anti-bacterial agents, outpatients, antibiotic optimization, antibiotic policy

## Abstract

“Antimicrobial stewardship” is a strategy that promotes the responsible use of antimicrobials. The objective of this paper is to focus on consolidated and more recent improvements in clinical strategies that should be adopted in hospitalized patients to ameliorate their infectious diseases’ outcome and to reduce the antibiotic resistance risk through judicious use of antibiotics. We present 13 common clinical scenarios, the respective suggested interventions and the explanations of the supporting evidence, in order to help clinicians in their decision-making process. Strategies including the choice of antibiotic and dose optimization, antibiotic spectrum narrowing (de-escalation), shortening of duration, shift to oral route or outpatient parenteral antibiotic (including elastomeric pumps), and biomarkers are described and discussed.

## 1. Introduction

Bacteria are continuously evolving and antibiotic resistances are significantly increasing, with 33,000 people in Europe, 35,000 in United States and >38,000 in Southeast Asia dying each year from antibiotic-resistant infections [1,2,3]. These numbers are expected to dramatically increase in the near future, with a worldwide estimate of 10 million deaths per year in 2050 due to antimicrobial resistance [4]. To limit the spread of antimicrobial resistance, reduce its related death and lower healthcare costs sustained for the management of drug resistant infections, the implementation of antimicrobial stewardship (AMS) programs has been advocated for several years. Since 20–50% of all antibiotics prescribed in acute-care hospital are either unnecessary or inappropriate [5,6], AMS has the potential capability to improve patient outcomes, reduce the incidence of antibiotic-related adverse events, curb antibiotic resistance and optimize resource utilization [7].

Despite AMS currently being an extensively used medical term, it lacks a universally endorsed definition. Many doctors believe that AMS sums up and embodies what both infectious diseases physicians and generally all antibiotic prescribers should do. Recently, Dyar et al. tried to provide a definition for AMS: the authors suggested “viewing AMS as a strategy, a coherent set of actions which promote using antimicrobials responsibly”, stressing the continuous need for “responsible use” to be defined and translated into context-specific and time-specific actions [8]. Several actions for AMS have been advocated and they include interventions both at a strategical level (e.g., education, restrictive and persuasive prescriptions) and at a patient level. For the latter, general principles have been extensively delineated, including recommendations on treating infections but not colonizations, implementing the appropriate use of antibiotics, narrowing broad-spectrum therapy, and selecting antibiotics with low *Clostridioides difficile* potential [9]. However, practical indications on how to implement AMS principles while taking care of individual patients at bedside are often lacking.

**Figure 1:**
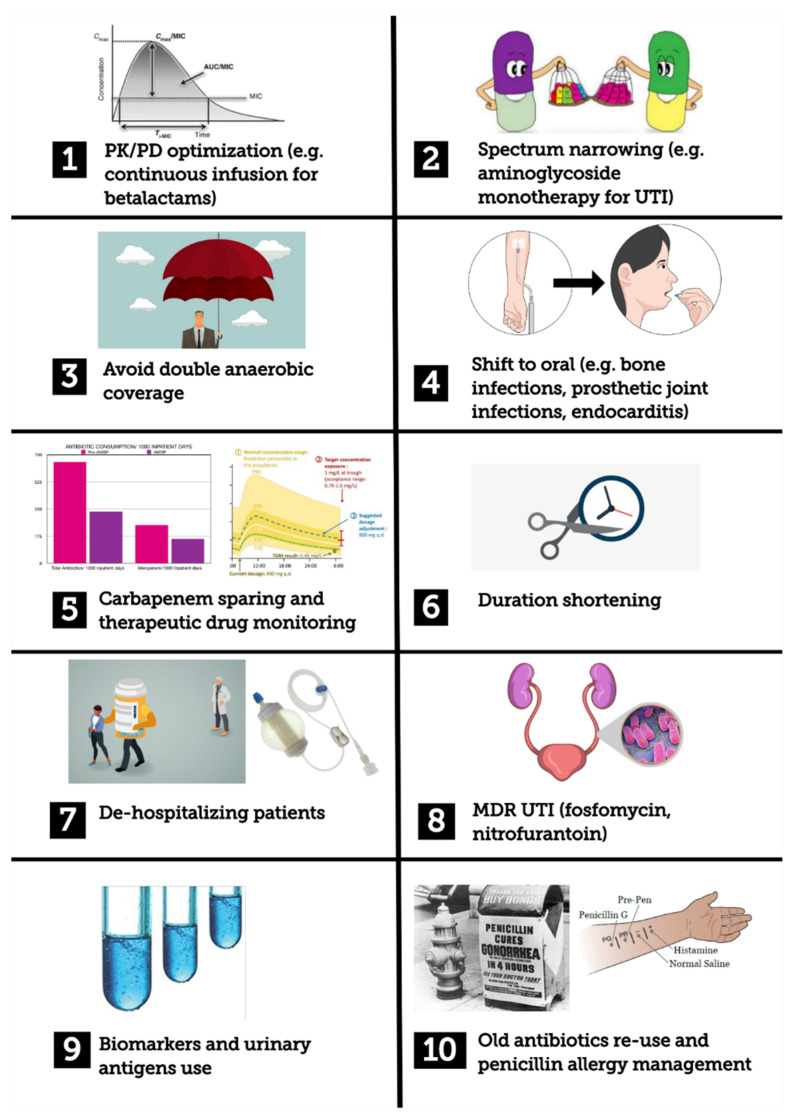
Graphical summary of stewardship interventions described in the manuscript. MDR: multidrug resistant; PK/PD: pharmacokinetic/pharmacodynamics; UTI: urinary tract infection

In light of these data and the given lack of pragmatism we found in several AMS papers, we tried to furnish common examples of clinical interventions that could help in both better understanding the AMS interventions by primary teams and improving the prescriptions themselves. On the basis of principles reported by guidelines and the current literature [7], and also on the basis of bedside clinical experience, we selected the following example scenarios: optimizing antibiotic dosing strategies, narrowing the antibiotic spectrum, avoiding unnecessary double anaerobic coverage, shift to an oral antibiotic regimen, shortening antibiotic duration, de-hospitalizing patients, approaching extended spectrum beta lactamases (ESBL) or carbapenem-resistant *Enterobacteriaceae* (CRE) urinary tract infections, using biomarkers and urinary antigens as stewardship tools, re-using old antibiotics, antibiotic allergy de-labelling (Figure 1). The choice of these clinical scenarios took into account the existing acute care antibiotic bundles [10] and what we believed to be significant situations faced during everyday clinical practice.

## 2. Thirteen Common Scenarios of Clinical AMS Strategies

### 2.1. Antibiotic Dosing Strategies

#### 2.1.1. Example 1

A 55-year old male patient with acute myeloid leukemia was treated with chemotherapy. He developed febrile neutropenia and sepsis. He had no central vascular catheter nor other obvious infectious focus. After blood cultures were drawn, an empiric therapy with intravenous piperacillin/tazobactam 4.5 g every 8 h was commenced by the hematologist.

#### 2.1.2. Intervention 1

Optimization of therapy was carried out by increasing the daily dose of piperacillin/tazobactam up to 18 g daily and administration of the antibiotic by continuous infusion.

#### 2.1.3. Comment 1

Neutropenic patients are at risk of severe infections especially due to Gram-negative organisms such as *Pseudomonas aeruginosa*, therefore the “anti-pseudomonas dosage” should be used (18 instead of 13.5 g). In addition, the literature data support the administration of beta-lactam antibiotics through continuous infusion for both increasing antibacterial activity and reducing sepsis-associated mortality [11].

### 2.2. Narrowing the Antibiotic Spectrum (Reducing the Induction of Multidrug-Resistant Bacteria and Clostridioides Difficile Infection) 

#### 2.2.1. Example 2

A 49-year old female obese patient, receiving insulin for diabetes mellitus, was admitted to the intensive care unit for septic shock and left leg ulcerative cellulitis. Empirical therapy with intravenous ceftriaxone was commenced. Two blood cultures yielded *Streptococcus pyogenes*.

#### 2.2.2. Intervention 2

Shift from intravenous ceftriaxone to intravenous penicillin G.

#### 2.2.3. Comment 2

Antibiotic therapy is a risk factor for developing infections from extended spectrum beta lactamases (ESBL)-producing bacteria and *C. difficile*. *C. difficile* has become the main cause of nosocomial infections in the western world [12]. Third generation cephalosporins are a strong risk factors for both extended spectrum beta-lactamases (ESBL)-producing microorganisms and *C. difficile* emergence [13,14]. In Example 2, shifting from ceftriaxone to penicillin G allows for narrowing the spectrum with a potent molecule but is poorly active against Gram negatives, thus potentially reducing the risk of developing ESBL and/or *C. difficile* infection. 

### 2.3. Unnecessary Double Anaerobic Coverage

#### 2.3.1. Example 3

A 67-year old male underwent a recent hemicolectomy for left colon cancer. Four days after surgery, he developed fever, tachypnea and altered mental status. No intra-abdominal collections were found by computed tomography scan. Empirical therapy with piperacillin/tazobactam and metronidazole was commenced. Following two days of antibiotic therapy, the patient clinically improved. No microbial growth was demonstrated in blood cultures.

#### 2.3.2. Intervention 3

Maintaining piperacillin/tazobactam while discontinuing metronidazole.

#### 2.3.3. Comment 3

Another frequently faced situation is the double anaerobic coverage in patients with intra-abdominal infections. Unnecessary double anaerobic coverage has been related to increased hospital costs, risk of drug-resistant pathogen acquisition, and the development of adverse reactions [15]. Almost half of the prescriptions of metronidazole in combination with other anti-anaerobic agents are unnecessary [15,16,17]. Since double anti-anaerobic coverage is a common scenario, it represents a relevant issue to be addressed by AMS efforts. The main antimicrobials with good anti-anaerobic coverage are shown in Table 1. Obtaining data from local antimicrobial susceptibility of anaerobes would be desirable prior to taking clinical decisions in this field.

### 2.4. Shift to Oral Antibiotic Regimen

#### 2.4.1. Example 4

A 39-year old female patient had native mitral valve endocarditis due to *Streptococcus mitis* showing penicillin minimum inhibitory concentration (MIC) ≤0.12 µg/mL. Her vital signs were stable and within normal limits. The echocardiographic examination showed mitral valve prolapse, evidence of an 8 × 13 mm vegetation, but no valve regurgitation or other abnormalities. No need for cardiac surgery was established. Targeted antibiotic therapy with ceftriaxone was started.

#### 2.4.2. Intervention 4

Fever disappeared at 36 h of antibiotic therapy and the patient’s vital signs remained stable. Therefore, after 10 days, antibiotic treatment was shifted from intravenous to oral regimen with amoxicillin 1 g orally every 6 h + rifampin 600 mg orally every 12 h.

#### 2.4.3. Comment 4

There is evidence that a shift from intravenous to oral antibiotic regimens in stable patients with left sided endocarditis is non-inferior to continued intravenous antibiotic treatment. The Danish trial published in 2018 included endocarditis due to *Streptococcus* spp., *Enterococcus faecalis*, *Staphylococcus aureus* or coagulase-negative staphylococci [18]. Patients with signs of abscess formation or valve abnormalities requiring surgery were excluded. The primary outcome was a composite of all-cause mortality, unplanned cardiac surgery, embolic events, or relapse of bacteremia with the primary pathogen. Patients were treated for at least 10 days (median 17 days) with intravenous therapy and then shifted to an oral regimen (Table 2) or maintained in intravenous therapy according to randomization. The primary composite outcome occurred in 12% of the intravenously treated group vs 9% of the orally treated group (*p* = 0.4) [18]. Prudence and commons sense are always welcome when facing endocarditis in order to not miss exclusion criteria (or complications) contraindicating the shift to oral antibiotic therapy. 

#### 2.4.4. Example 5

A 55-year old man with persistent fever and lumbar pain was diagnosed with lumbar vertebral osteomyelitis (L4-L5). No neurological abnormalities were found. Diagnosis of probable vertebral osteomyelitis (L4-L5) was obtained by magnetic resonance imaging. The tissue culture from bone biopsy grew methicillin-susceptible S. aureus. Intravenous therapy with oxacillin 2 g every 4 h was commenced.

#### 2.4.5. Intervention 5

After 7 days, the intravenous antibiotic therapy was changed to an oral regimen including levofloxacin 500 mg every 12 h plus rifampicin 450 mg every 12 h. 

#### 2.4.6. Comment 5

The shift from intravenous to oral antibiotic therapy in bone and joint infections has been associated with a shorter length of hospital stay and fewer complications. In the trial by Li et al. published in 2019, there were no differences in terms of treatment failure in patients with bone or joint infection shifted to oral therapy within 7 days after surgery (or, if the infection was being managed without surgery, within 7 days after the start of antibiotic treatment) vs those who continued intravenous antibiotic treatment [19]. In fact, treatment failure occurred in 14.6% of the intravenous group and 13.2% of the oral group. No difference in serious adverse events between the two groups was noticed, but catheter complications were more common in the intravenous group (9.4% vs 1%) [19]. A shift from intravenous to oral administration turns to be one of the main topics of AMS. Indeed, this policy allows the removal of vascular catheters lowering catheter-associated complications and encouraging prompt discharge improving patients’ quality of life and reducing health-care costs.

### 2.5. Shortening Antibiotic Duration

#### 2.5.1. Example 6

Bacteremic urosepsis due to Escherichia coli was diagnosed in a 65-year-old male with an indwelling urinary catheter for benign prostate hyperplasia. The patient developed fever and chills without other symptoms; his vital signs were stable and within normal limits. Bacteremic urosepsis due to E. coli was diagnosed and the urinary catheter was promptly replaced, and intravenous antibiotic therapy with ceftriaxone was administered, leading to defervescence, and the fever disappeared after 48 h of treatment.

#### 2.5.2. Intervention 6

Stop antibiotic therapy after 7 days of treatment.

#### 2.5.3. Comment 6

A recent trial compared a 7-day vs 14-day course of antibiotic therapy for Gram-negative bacteremias. Patients who were afebrile and hemodynamically stable for at least 48 h were randomized in two arms to receive 7 or 14 days of intravenous antibiotics. The primary outcome at 90 days was a composite of all-cause mortality, relapse, suppurative, or distant complications, and readmission or extended hospitalization (>14 days). Patients with uncontrolled source of infection were excluded, as well as neutropenic, transplanted or HIV-seropositive. There were no significant differences in the primary outcome between the two groups [20].

#### 2.5.4. Example 7

A diabetic 70-year-old female was diagnosed with native joint arthritis of her left knee. A synovial fluid culture grew methicillin-susceptible S. aureus. Intravenous oxacillin was commenced and surgical drainage was performed. 

#### 2.5.5. Intervention 7

Stop antibiotic therapy after a 2-week course of therapy following successful surgical drainage.

#### 2.5.6. Comment 7

A trial published in 2019 compared 77 patients treated with a 2-week course with 77 patients treated with 4-week course of antibiotic therapy [20]. The week count was considered after surgical drainage (episodes without surgical lavage were excluded). A minimal follow-up of 2 months was ensured. No difference in cure rate, recurrences and sequelae was found between the two groups [21]. Nowadays, it is possible to shorten the duration of antibiotic therapy even for other common indications, including community-acquired pneumonia (5–7 days) [22,23], ventilator-associated pneumonia (VAP) (8 days) [24], uncomplicated pyelonephritis (5–7 days if treated with a fluoroquinolone) [25], complicated intra-abdominal infections (5 days after adequate source control) [26]. Shorter therapy duration was found to be associated with the emergence of fewer (−20%) multidrug resistant pathogens [24]. 

### 2.6. De-Hospitalizing Patients

#### 2.6.1. Example 8

A 66-year-old male, affected by chronic obstructive pulmonary disease, was diagnosed with fever, worsening of cough and chest pain on the left side. His past medical history was remarkable for chronic obstructive pulmonary disease. A chest X-ray showed a left upper lobe infiltrate consistent with a lung abscess. A sputum culture demonstrated *P. aeruginosa* resistant to ciprofloxacin. Intravenous antibiotic therapy with piperacillin/tazobactam was commenced, with improvement in the patient’s clinical conditions and normalization of vital signs within 3 days.

#### 2.6.2. Intervention 8

On day 3 of antibiotic therapy, the patient’s clinical conditions greatly improved and his vital signs returned to normal limits. On day 7, a midline catheter was inserted and the patient was discharged. He was referred to an outpatient service to continue continuous infusion of piperacillin/tazobactam (18 g daily), administered through an elastomeric pump, for 5 weeks. 

#### 2.6.3. Comment 8

There is growing evidence that infusion pump (e.g., elastomeric pumps) use is an effective outpatient parenteral antibiotic therapy strategy to facilitate de-hospitalization, especially in patients treated with time-dependent antibiotics [27]. When antibiotics are delivered through elastomeric pumps, the individuals should be visited on an outpatient basis once a day for receiving the antibiotic solution reconstituted, bringing the infusion pump in a pouch. Indeed, this policy improves patient quality of life, allowing the possibility to continue normal life activities with scarce limitations. The use of elastomeric pumps cannot be completely assimilated to continuous infusions in hospitalized patients because of temperature issues (pumps are close to patients’ skin), device materials and forced dilutions that could affect antibiotic stability. Moreover, the use of outpatients’ infusion pumps requires at least a medium-term peripheral venous access (e.g., midline catheter). However, most antibiotics administered in continuous infusion in hospitalized patients also have evidence-based data from studies conducted with elastomeric pumps (Table 3). Before starting beta-lactams and vancomycin infusion, it is advisable to administer a loading dose in order to reduce time to reach target concentrations [28,29]. A list of antibiotics suitable for continuous infusion is furnished in Table 3.

### 2.7. ESBL or Carbapenem-Resistant Enterobacteriaceae (CRE) Urinary Tract Infections

#### 2.7.1. Example 9

A 74-year old man with relapsing urinary tract infections and benign prostatic hyperplasia was scheduled for transurethral biopsy of the prostate. One month before, he was treated for urinary tract infection; at that time, the urine culture grew 100,000 CFU/mL of ESBL-producing *E. coli*. 

#### 2.7.2. Intervention 9

Fosfomycin trometamol 3 g *per os* given once before bedtime the day before prostate biopsy was prescribed. 

#### 2.7.3. Comment 9

Fosfomycin trometamol has an excellent urinary concentration with mean peak urinary concentrations (within 4 h from administration) ranging from 1053 to 4415 mg/L after a single 3 g dose [56]. Such a high concentration is multiple times above the minimal inhibitory concentration of most urinary pathogens, making fosfomycin a good option for urinary tract infections, and also for those sustained by multi-drug resistant pathogens.

A recent systematic review of the studies on fosfomycin in comparison with fluoroquinolones to prevent trans-rectal ultrasound-guided prostate biopsy-related infectious complications showed that patients who received fosfomycin prophylaxis were less likely than those who received fluoroquinolone prophylaxis to develop infections overall, as well as severe and resistant infections. The difference was explained by fluoroquinolone resistance and ESBL production in pathogens causing post-biopsy infections. A retrospective study on 52 patients with urinary tract infections from ESBL-producing *E. coli* treated with fosfomycin trometamol 3 g *per os* every 48 h 3 times reported a clinical and microbiological success rate of 94% and 78%, respectively [57].

Apart from ESBL-producing bacteria, fosfomycin has been demonstrated to be a viable option for infections sustained by CRE. In fact, fosfomycin is active against approximately 80% of CRE, especially *Klebsiella pneumoniae* carbapenemase (KPC)-producing *Klebsiella pneumoniae*, including colistin-resistant strains [58], and represent an helpful drug from an AMS point of view.

### 2.8. Biomarkers and Urinary Antigens as Stewardship Tools

#### 2.8.1. Example 10

A 65-year-old female patient with no previous medical history was diagnosed with community-acquired pneumonia. Intravenous ampicillin/sulbactam and clarithromycin were commenced. Urinary pneumococcal antigen tested positive.

#### 2.8.2. Intervention 10

Clarithromycin was discontinued and ampicillin/sulbactam de-escalated to penicillin G.

#### 2.8.3. Comment 10

Macrolides are overprescribed antibiotics and represent an easy target for AMS interventions. It has been demonstrated that the hospital rate of pneumococcal urinary antigen testing was strongly correlated with de-escalation following a positive test [59], suggesting that the use of urinary antigen testing could also be enforced in terms of an AMS viable tool. Regarding *Streptococcus pneumoniae* pneumonia, de-escalation to penicillin G is safe when penicillin G MIC in pneumococci ≤2 µg/mL (again we recommend relying on reliable local or national susceptibility testing data if the bacterial isolate has not grown).

#### 2.8.4. Example 11

A 50-year-old male affected by obesity and diabetes mellitus was admitted to an intensive care unit for sepsis with severe respiratory insufficiency. A diagnosis of influenza complicated by bacterial pulmonary superinfection due to *Haemophilus influenzae* was established. Procalcitonin on diagnosis was 10 µg/L (normal values <0.5 µg/L). After 5 days of oral oseltamivir and intravenous ampicillin/sulbactam, the patient experienced significant clinical amelioration and procalcitonin dropped to 0.5 µg/L.

#### 2.8.5. Intervention 11

Antibiotic therapy was stopped after 5 days.

#### 2.8.6. Comment 11

Procalcitonin has been used as a tool to support clinical decisions on antibiotic discontinuation. A prospective, multicenter, randomized, controlled, open-label intervention trial compared two strategies in Dutch intensive care units. The authors included 761 patients in the procalcitonin-guided group and 785 patients in the standard-of-care group. In the procalcitonin-guided group, non-binding advice to discontinue antibiotics was provided if procalcitonin concentration had decreased by 80% or more of its peak value or to 0.5 µg/L or lower. The results show that the median duration of treatment was 5 days in the procalcitonin-guided vs 7 days in the standard-of-care-guided group (*p* < 0.0001) and this was also associated with a significantly reduced mortality in the procalcitonin-guided group (20% vs 27% [*p* = 0.0154] in the per-protocol analysis) [60].

### 2.9. Old Antibiotics Reuse

#### 2.9.1. Example 12

A 70-year-old man with history of nephrolithiasis and benign prostatic hyperplasia developed dysuria and high-grade fever accompanied by shaking chills. Urine culture grew 10^6^ CFU/mL of ESBL-producing *K. pneumoniae*. The strain was resistant to piperacillin/tazobactam (MIC 32 µg/mL), and fosfomycin but susceptible to temocillin (MIC 4 µg/mL).

#### 2.9.2. Intervention 12

Intravenous temocillin: 2 g loading dose followed by 6 g in 24 h continuous infusion was started with progressive amelioration of clinical signs and symptoms and improvement of biochemical parameters.

#### 2.9.3. Comment 12

Temocillin is a carboxypenicillin active against ESBL, KPC and AmpC-producing *Enterobacteriaceae*. It is a forgotten antibiotic, recently rediscovered and used for bacteremia, urinary tract infection and lower respiratory tract infection. The cumulative percentage of a 24 h period during which the free drug concentration exceeds the minimum inhibitory concentration (fT > MIC) is the best pharmacokinetic/pharmacodynamic index [61]. The data showed that continuous infusion allows one to better target attainment compared to intermittent administration [54]. Temocillin is not commonly available in most developed countries, therefore we encourage efforts to procure/import the drug.

### 2.10. Antibiotic Allergy De-Labelling

#### 2.10.1. Example 13

A 75-years old man underwent the removal of a shoulder prosthesis for a prosthetic joint infection six months previously. His postoperative course seemed to be uneventful, but he did not stop complaining of stress-induced and nocturnal resting pain, and poor mobility. Revision was performed with the removal of the prosthesis. Sonication fluid grew *Cutibacterium acnes* 100 CFU/mL, and three out of four additional bone samples were positive for the same microorganism. The patient’s medical history revealed an allergy to penicillin. The patient was started on ceftriaxone 2 g every 24 h intravenously.

#### 2.10.2. Intervention 13

The patient was sent to allergology clinics and the immediate type of allergy to penicillin was excluded by skin test. After 14 days of parenteral therapy, the patient was switched to oral treatment with amoxicillin 1000 mg every 8 h for additional 4 weeks.

#### 2.10.3. Comment 13

Antibiotic allergy labels that reflect variable adverse drug reactions are found in 10–30% of hospitalized patients. The labels lead to the substitution of antibiotics of choice with second line drugs that are often associated with microbial resistance, higher cost or even sub-optimal patient outcomes. Antibiotic allergy in-depth review has been shown to remove the label in more than 90% of cases, and may contribute to better antibiotic use [62]. De-labelling has been recommended as an antimicrobial stewardship strategy in Infectious Diseases Society of America guidelines [7].

## 3. Discussion 

Antibiotic misuse or overuse is still highly prevalent in the hospital setting. Indeed, previous reports have shown that infectious disease specialist consultation can lead to resource-saving advices in more than 40% of cases [6]. The implementation of AMS programs has been advocated in order to counteract the increase in bacterial resistance and its related consequences [7]. While the general principles of AMS have long since been established, AMS programs are continuously evolving. This is partly a consequence of evolving scientific knowledge and the implementation of novel approaches to patient care. On the other hand, there are difficulties in increasing AMS programs’ efficiency due to a gap between the theoretical benefits of selected interventions and their implementation in real life. An important limitation is given by the fact that AMS programs address general principles that may not be generalizable in all settings.

In our manuscript, we have described several clinical scenarios frequently occurring during routine clinical practice, and we have proposed targeted practical interventions to optimize antibiotic therapy. Each scenario elucidates how the main principles of AMS could be applied at a patient level. Narrowing the antibiotic spectrum (intervention 2) is the mainstay of correct antibiotic usage and, when correctly applied, has the potential to obtain clinical success while minimizing the risk of increasing resistance. It is strictly dependent on microbiological data such as a continuous monitoring of local resistance epidemiology, but also the development of methods for accurate and rapid identification of pathogens and characterization of their resistance profile [63]. Urinary antigen use could be useful for this purpose (intervention 10). Interestingly, narrowing the antibiotic spectrum can also be obtained in the management of selected uncomplicated infections caused by multi-drug-resistant (MDR) pathogens, such as ESBL or CRE urinary tract infections (intervention 9). In the setting of MDR, the re-use of old antibiotics should also be considered (intervention 12).

The optimal use of antibiotics can also be obtained by the optimization of dosage or administration modes on the basis of their pharmacokinetic/pharmacodynamics properties (intervention 1). A shift from intravenous to oral therapy when the patient is stable (interventions 4–5), especially to drugs with high oral bioavailability [64], is one of the interventions endorsed in AMS programs. Recently, a rapid shift to oral antibiotics has been proposed also for the management of selected endocarditis and bone/joints infections [18,19], conditions traditionally treated with long cycles of parenteral therapy. This approach can reduce the risk of phlebitis, intravenous line-associated infections and length of hospital stay. The latter can also be obtained by shortening antibiotic duration in scenarios characterized by rapid clinical response and source control (interventions 6–7), or with the aid of biomarker monitoring (i.e., procalcitonin) (intervention 11). In patients requiring long term antibiotic treatment, de-hospitalizing patients can be pursued by implementing outpatient antibiotic therapy services (intervention 8).

AMS programs should also take into account all scenarios characterized by antibiotic misuse. The double anaerobic coverage (intervention 3) is frequently observed in the management of intra-abdominal infections and should generally be avoided. Moreover, in patients with reported penicillin allergy, de-labelling is preferred over using a second-line drug, which is often a broad spectrum antibiotic with a higher impact on resistance spread (intervention 13).

## 4. Conclusions

In conclusion, we provided a series of real-life clinical scenarios in order to discuss the main current topics of AMS. The aim of the present manuscript was to furnish practical cues for physicians involved in the management of bacterial infections. Some of the interventions described are already well established and supported by a large volume of evidence, while the others are based on recently published studies and look promising for at least some patients. The evidence is expected to change over the time, and, to improve antibiotic prescribing, we need to include it meaningfully in our clinical practice. There is a role for AMS teams to introduce new evidence and promote the use of well-established practices, but at the same time every prescribing physician should improve his knowledge on up-to date antibiotic treatments in his field of expertise.

## Figures and Tables

**Table 1:** Commonly used antibiotics with anaerobic coverage.

	Degree of Activity	
Antibiotics with Anaerobic Coverage	Beta-Lactamase Producing Anaerobes	Other Anaerobes
Amoxicillin/clavulanate	+++	+++
Cefoxitin	++	+++
Chloramphenicol	+++	+++
Clindamycin	++	+++
Ertapenem	+++	+++
Imipenem	+++	+++
Meropenem	+++	+++
Metronidazole	+++	+++
Moxifloxacin	++	++
Penicillin	0	+++
Piperacillin/tazobactam	+++	+++
Ticarcillin	+	+++
Tigecycline	++	+++

Adapted from Brook et al. [15].

**Table 2:** Oral antibiotic regimens adopted in the Danish trial [18].

Penicillin and methicillin sensitive S. *aureus* and coagulase-negative staphylococci	amoxicillin 1 g q6 h and fusidic acid 750 mg q12 h
	amoxicillin 1 g q6 h and rifampicin 600 mg q12h
	linezolid 600 mg q12 h and fusidic acid 750 mg q12 h
	linezolid 600 mg q12 h and rifampicin 600 mg q12 h
Methicillin sensitive S. *aureus* and coagulase-negative staphylococci	dicloxacillin 1 g q6 h and fusidic acid 750 mg q12 h
	dicloxacillin 1 g q6 h and rifampicin 600 mg q12 h
	linezolid 600 mg q12 h and fucidic acid 750 mg q12 h
	linezolid 600 mg q12 h and rifampicin 600 mg q12 h
Methicillin resistant coagulase-negative staphylococci	linezolid 600 mg q12 h and fusidic acid 750 mg q12 h
	linezolid 600 mg q12 h and rifampicin 600 mg q12 h
*E. faecalis*	amoxicillin 1 g q6 h and rifampicin 600 mg q12 h
	amoxicillin 1 g q6 h and moxifloxacin 400 mg q24 h
	linezolid 600 mg q12 h and rifampicin 600 mg q12 h
	linezolid 600 mg q12 h and moxifloxacin 400 mg q24 h
Streptococci with a penicillin MIC < 1 mg/L	amoxicillin 1 g q6 h and rifampicin 600 mg q12 h
	linezolid 600 mg q12 h and rifampicin 600 mg q12 h
	linezolid 600 mg q12 h and moxifloxacin 400 mg q24 h
Streptococci with penicillin MIC ≥ 1 mg/L	linezolid 600 mg q12 h and rifampicin 600 mg q12 h
	moxifloxacin 400 mg q24 h and rifampicin 600 mg q12 h
	moxifloxacin 400 mg q24 h and clindamycin 600 mg q8 h

MIC: minimal inhibitory concentration.

**Table 3:** Common antimicrobials used for continuous infusion.

Antibiotic	Stability at 25 °C	Diluent	Existing Data in Elastomeric Pumps	References
amoxicillin	12 h	WFI/NS	Yes	[30]
ampicillin	30 h	NS	Yes but conflicting	[31]
aztreonam	48	D5W	Yes	[32]
Cefazolin *	24 h	NS/D5W	Yes	[33,34,35]
cefepime	24 h	NS	Yes	[35,36]
Cefotaxime *	12–24 h	NS/D5W	No	[37,38]
cefoxitin	48 h	NS/D5W	Yes	[39]
ceftaroline	24 h	NS/D5W	Yes	[40]
ceftazidime	48 h	NS	Yes	[33,41]
ceftazidime/avibactam	12 h	NS/D5W/RL	Yes	[42]
ceftolozane/tazobactam	24 h	NS/D5W	Yes	[43]
cefuroxime	48 h	NS	Yes	[44,45]
clindamycin	16 d	D5W	No	[46]
flucloxacillin	24 h	NS	Yes	[35,47]
Fosfomycin *	24 h	WFI	No	[48,49]
meropenem	4 h	NS	Unsuitable (short stability)	[50]
oxacillin	24 h	NS	No	[51]
benzylpenicillin potassium	24–48 h	RA	Yes	[33,52]
benzylpenicillin sodium	12–24 h	NS	Yes	[53]
piperacillin/tazobactam	24 h	NS	Yes	[35]
temocillin	24 h	WFI	Yes	[54,55]
vancomycin	7 d	NS	Yes	[27]

* protect the reconstituted solution from sunlight; EP: elastomeric pumps; NS: normal saline; D5W: dextrose 5% in water; RA: ringer acetate; RL: ringer lactate; WFI: water for injection.
